# Endocystectomy as a conservative surgical treatment for hepatic cystic echinococcosis: A systematic review with single-arm meta-analysis

**DOI:** 10.1371/journal.pntd.0009365

**Published:** 2021-05-12

**Authors:** Mohammad Al-Saeedi, Ali Ramouz, Elias Khajeh, Ahmad El Rafidi, Omid Ghamarnejad, Saeed Shafiei, Sadeq Ali-Hasan-Al-Saegh, Pascal Probst, Marija Stojkovic, Tim Frederik Weber, Katrin Hoffmann, Arianeb Mehrabi

**Affiliations:** 1 Department of General, Visceral, and Transplantation Surgery, University of Heidelberg, Heidelberg, Germany; 2 Section of Clinical Tropical Medicine, University Hospital Heidelberg, Heidelberg, Germany; 3 Department of Diagnostic and Interventional Radiology, University Hospital Heidelberg, Heidelberg, Germany; 4 Liver Cancer Center Heidelberg, Heidelberg University Hospital, Heidelberg, Germany; University of Zurich, SWITZERLAND

## Abstract

**Background:**

In patients with hepatic cystic echinococcosis (CE), treatment effectiveness, outcomes, complications, and recurrence rate are controversial. Endocystectomy is a conservative surgical approach that adequately removes cyst contents without loss of parenchyma. This conservative procedure has been modified in several ways to prevent complications and to improve surgical outcomes. This systematic review aimed to evaluate the intraoperative and postoperative complications of endocysectomy for hepatic CE as well as the hepatic CE recurrence rate following endocystectomy.

**Methods:**

A systematic search was made for all studies reporting endocystectomy to manage hepatic CE in PubMed, Web of Science, and Cochrane CENTRAL databases. Study quality was assessed using the methodological index for non-randomized studies (MINORS) criteria and the Cochrane revised tool to assess risk of bias in randomized trials (RoB2). The random-effects model was used for meta-analysis and the arscine-transformed proportions were used to determine complication-, mortality-, and recurrence rates. This study is registered with PROSPERO (number CRD42020181732).

**Results:**

Of 3,930 retrieved articles, 54 studies reporting on 4,058 patients were included. Among studies reporting preoperative anthelmintic treatment (31 studies), albendazole was administered in all of them. Complications were reported in 19.4% (95% CI: 15.9–23.2; I^2^ = 84%; p-value <0.001) of the patients; biliary leakage (10.1%; 95% CI: 7.5–13.1; I^2^ = 81%; p-value <0.001) and wound infection (6.6%; 95% CI: 4.6–9; I^2^ = 27%; p-value = 0.17) were the most common complications. The post-endocystectomy mortality rate was 1.2% (95% CI: 0.8–1.8; I^2^ = 21%; p-value = 0.15) and the recurrence rate was 4.8% (95% CI: 3.1–6.8; I^2^ = 87%; p-value <0.001). Thirty-nine studies (88.7%) had a mean follow-up of more than one year after endocystectomy, and only 14 studies (31.8%) had a follow-up of more than five years.

**Conclusion:**

Endocystectomy is a conservative and feasible surgical approach. Despite previous disencouraging experiences, our results suggest that endocystectomy is associated with low mortality and recurrence.

## Author summary

Cystic echinococcosis (CE) is a parasitic zoonosis caused by the larval stage of *Echinococcus granulosus sensu lato*, which can invade several organs including the brain, lungs, and liver. Liver involvement through hepatic cystic echinococcosis has been reported in up to 75% of CE cases. Although it is a rare disease in Europe, migrations from endemic regions to European countries have increased the prevalence of hepatic CE. Several medical and surgical treatment options have been introduced for hepatic CE. Of these, endocystectomy is a conservative approach which avoids radical surgeries and liver resections. In this systematic review and single-arm meta-analysis, we synthesized evidence on the short- and long-term outcomes of endocystectomy in treating patients with hepatic CE. We searched for relevant articles in PubMed, Embase, and Cochrane databases that were published until May 2020 without any language restriction. Of 3930 articles, 54 were included in the systematic review. A summarized analysis of the study findings suggested that endocystectomy is an effective and safe alternative to radical resection or the PAIR procedure in hepatic CE patients.

## Introduction

Hepatic cystic echinococcosis (CE) is a parasitic zoonosis caused by the larval stage of *Echinococcus granulosus sensu lato* [1, 2]. Cystic lesions occur in the liver in up to 75% of CE cases. Despite being endemic in certain regions, hepatic CE is rare in most developed countries [3]. However, the incidence of CE has increased in Europe because of inward migration from endemic areas [4, 5]. It has now become important to determine the best treatment options for these patients in European countries.

Hepatic CE can be treated with observational, medical, percutaneous, and surgical approaches. The World Health Organization (WHO) Informal Working Group on Echinococcosis has recommended defining the treatment strategy based on the cyst stage [6]. Accordingly, patients with cysts greater than 10 cm or in CE stages 2 or 3b (with daughter cysts) should be treated surgically [6]. However, other cysts may also require immediate surgical treatment, such as superficial cysts with a higher risk of rupture, infected cysts, or cysts communicating with the biliary tree [6].

Surgery is considered the treatment of choice for patients with large cysts and complicated hepatic CE because satisfying outcomes have been reported [7–9]. Endocystectomy is a conservative non-resectional surgical procedure that adequately removes cyst contents. Since it was first introduced by Lindeman in 1871, endocystectomy has been modified several times to reduce complications and recurrence, and to improve outcomes. These modifications include endocystectomy with external drainage, omentoplication, and capitonnage. However, removal of the parasitic germinal layer remains a problem and increases the chance of recurrence.

The current systematic review aimed to evaluate the intraoperative and postoperative complications of endocystectomy for hepatic CE and hepatic CE recurrence rate after endocystectomy.

## Methods

This study is reported according to the Preferred Reporting Items for Systematic Reviews and Meta-Analyses (PRISMA) statement [10].

### Search strategy and selection criteria

A systematic literature search in Medline (via PubMed), Cochrane CENTRAL, and ISI Web of Science was conducted using a combination of the following search terms: “((liver[Title/Abstract] OR hepatic[Title/Abstract])) AND (surgical[Title/Abstract] OR surgery[Title/Abstract] OR operative[Title/Abstract] OR operation[Title/Abstract])) AND (echinococcus[Title/Abstract] OR echinococcosis[Title/Abstract] OR hydatid cyst[Title/Abstract] OR hydatidosis[Title/Abstract])” [11]. The search was not restricted to a specific study type or year of publication. The last search was performed in May 2020.

The study question was formulated based on the Population, Intervention, Comparison, Outcome, and Study design (PICOS) strategy. Studies were included if they met the following criteria:

- *Population*: Patients with hepatic CE who underwent endocystectomy.
- *Intervention*: Endocystectomy, regardless of accompanied external drainage, omentoplication, pericystectomy, or capitonnage.
- *Comparison*: None.
- *Outcome*: Relevant preoperative data, intra- and postoperative complications, and disease recurrence.
- *Study design*: All study designs were eligible for inclusion.

Experimental studies, letters, comments, and editorials were excluded. We also cross-checked for reviews and double publications to avoid including the same data more than once. The reference lists of the retrieved articles were screened for additional relevant studies.

### Study selection and data extraction

The titles and abstracts of the retrieved articles were independently screened by two authors (EK and AR). The full articles of interest were then reviewed by two other authors (OG and SS) to select articles suitable for data extraction. In case of disagreement, the senior author (AM) decided to include or exclude a study. Data were extracted by two reviewers independently. Extracted items were study characteristics (year of the study, number of cases, country, and study design), clinical presentation, cyst classification, preoperative diagnostic workups, perioperative medical treatments, surgical techniques, intra- and postoperative complications, mortality, and recurrence rate.

This systematic review is registered in the PROSPERO International Prospective Register of systematic reviews under the registration number CRD42020181732 (https://www.crd.york.ac.uk/PROSPERO/display_record.php?RecordID=181732).

### Quality assessment

The quality of each study was assessed by two independent reviewers using the MINORS criteria [12]. Quality of non-comparative studies was determined based on the first eight items; the last four items were only used to assess comparative studies (S1.a Table). For each item, the scoring system was: 0 (not reported), 1 (reported but inadequate), or 2 (reported and adequate). For non-comparative studies, an overall score of more than 12 points indicated high quality; between 8 and 12 points indicated intermediate quality; and less than 8 points indicated low quality. For comparative studies, the highest possible score was 24. A score of more than 18 points indicated high quality; 12–18 points indicated intermediate quality; and less than 12 points indicated low quality. To evaluate the methodological quality of randomized controlled trials (S1.b Table), the Cochrane Risk of Bias Tool for Randomized Controlled Trials 2.0 was used [13]. This tool evaluated five essential items: 1) bias arising from the randomization process, 2) bias arising from the timing of identification and recruitment of individual participants in relation to the timing of randomization, 3) bias due to deviations from intended interventions, 4) bias due to missing outcome data, and 5) bias in selecting the reported results. These domains were rated as “high risk of bias”, “low risk of bias”, or “some concerns”. Finally, an overall risk of bias was determined. The overall risk of bias was “high risk of bias” if at least one domain was deemed “high risk of bias” or if there were “some concerns” in three or more domains. The overall risk of bias was “some concerns” if there was “some concerns” in at least one domain. The overall risk of bias was “low risk of bias” if all domains were rated as “low risk of bias”.

### Definition of extracted data

#### Demographic and baseline characteristics

General study information, including year, number of cases, country, and study design were collected. Hepatic CE manifestation at preoperative presentation, including clinical signs and symptoms, were qualitatively extracted. Preoperative diagnostics included laboratory tests and imaging. Laboratory tests included the Casoni test, assays providing an antibody titer, and an ELISA assay. Imaging techniques included plain chest and abdominal X-ray radiography, ultrasonography, computed tomography (CT) scan, and magnetic resonance imaging (MRI). Preoperative interventions, including magnetic resonance cholangiopancreatography (MRCP) and endoscopic retrograde cholangiopancreatography (ERCP), were recorded. Cyst stages were determined based on the reported ultrasonographic evaluation in each study. The studies used either the WHO [6] or Gharbi et al. [14] classifications. Cysts in stages CE3 were reported according to the data of original studies. In studies that distinguished between CE3a and CE3b cysts, data on the two types were reported separately, and in studies reporting overall CE3 cysts, data on CE3 cysts were reported with no further classification. We also recorded whether preoperative anthelmintic treatments such as albendazole and mebendazole or a mixed medical treatment approach were administered. The treatment period and administration schedule (continuous or cyclic) were also recorded.

#### Surgical technique

We recorded whether the surgery was laparoscopic or open. We also recorded additional details of the surgical intervention, such as modifications, type of scolicidal agent applied, duration of solicidal agent contact with the germinal layer, and the number of application attempts. We also noted the technique used to manage the residual cystic cavity, such as omentoplasty, marsupialization, drainage, capittonage, diaphragm myoplasty, introflextion, and hepatoplasty. Marsupialization involved cutting a slit into the cyst and suturing the edges to form a continuous surface. Capitonnage included suturing the edges of the remnant anterior cystic wall to the posterior wall. In omentoplasty, part of the greater omentum was used to cover or fill the residual cavity.

#### Intraoperative complications

Intraoperative complications were recorded as anaphylactic shock and other complications, including cyst contents spillage, hemorrhage, bile leakage, and iatrogenic injury of other organs.

#### Postoperative complications and mortality

Postoperative complications were morbidities that occurred after the surgical intervention, such as biliary leakage, biliary fistula, residual cavity bleeding, residual cavity infection, pleural effusion, wound infection, anemia, and pneumonia. The complications were reported according to the Clavien–Dindo classification, where data were provided [15]. Postoperative mortality was defined as all endocystectomy-related death events occurring within 90 days of endocystectomy.

#### Follow-up and recurrence

Data regarding the duration of follow-up after endocystectomy and anthelmintic prophylaxis were gathered. Postoperatively, the type of medication, treatment period, and administration schedule (continuous or cyclic) were recorded. Patients who were lost to follow-up after endocystectomy were also reported. Hepatic CE recurrence was defined as all recurring cysts, not just those detected in the liver.

### Statistical analysis

Meta-analyses were conducted according to statistical heterogeneity between the studies using Open MetaAnalyst Software version for Mac. The random-effects model was used for meta-analysis and the arscine-transformed proportions were used to determine complication-, mortality-, and recurrence rates. The total clinical setting percentage for the main outcome and number of participants of each study were input to calculate the corresponding standard errors of these quasinormal distribution “rates”. The 95% confidence lower interval and upper interval derived from the “rates” and standard errors could be justified. Lastly, the pooled effect sizes, which denoted median “rates” and the 95% confidence intervals (95% CI), were output. Statistical heterogeneity was explored with the Chi-square test. Heterogeneity among effect sizes of individual studies was assessed using the I^2^ index and Q statistic. Heterogeneity was analyzed with the I^2^ statistic and was defined as low (25% to 50%), moderate (50% to 75%), or high (> 75%).

The subgroup analyses of the outcomes based on surgical modification of the residual cavity management and surgical approach (open and laparoscopic) were performed using the Chi-square test for categorical data. A p-value < 0.05 was considered statistically significant. While comparing the surgical modifications of residual cavity management, a post-hoc analysis was carried out by calculating the standardized residuals of crosstabulation ((frequency-expected)/standard error) if the Chi-square test gave a significant result. The two-sided α level was adjusted by Bonferroni correction for multiple comparisons.

## Results

### Study characteristics

**Fig 1:**
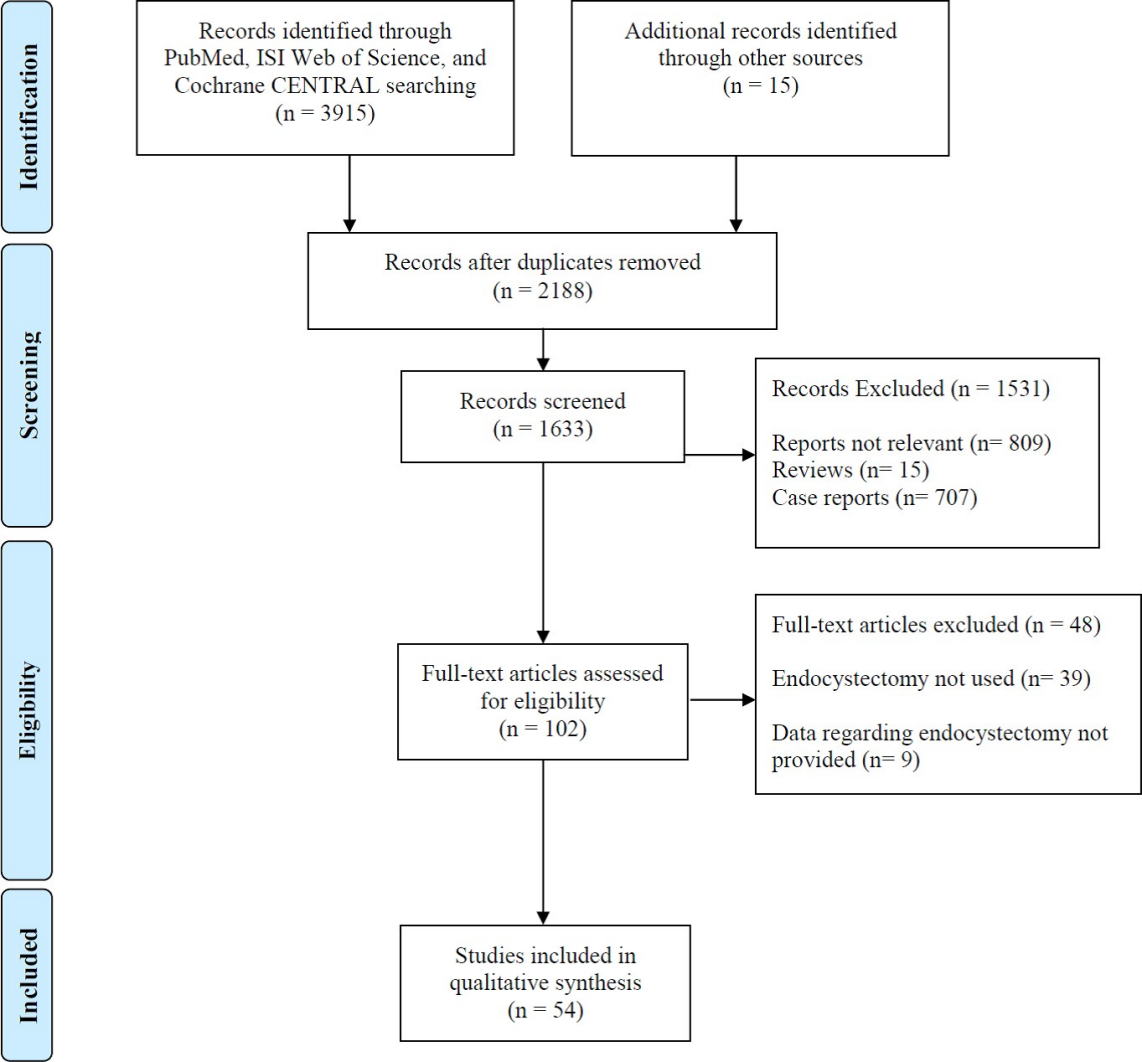
PRISMA flow-chart showing selection of articles for review.

The initial database searches yielded 3,930 articles, as shown in the PRISMA flow diagram in Fig 1. After initial screening, 1,742 papers were removed because of data duplication. After the application of inclusion and exclusion criteria, 54 articles with 4,058 patients were selected for review. These studies were published between 1991 and 2019; 33 studies (66.1%) were retrospective; 19 studies (35.2%) were prospective, and two studies (3.7%) were randomized controlled trials. The included studies are summarized in Table 1.

**Table 1:** Study design, sample size, surgical technique, and residual cavity management.

Author (year)	Country	Number of cases	Study design	Open/laparoscopic surgery	Residual cavity management			
					Omentoplasty	Marsupilarization	Capitonnage	Drain
Bayrak (2019)[51]	Turkey	29	Retrospective	Open and laparoscopic	29	-	-	-
Al-Saeedi (2019)[5]	Germany	21	Retrospective	Open	14	-	-	-
Magistri (2019)[52]	Italy	5	Retrospective	Open	-	-	-	-
Marom (2019)[53]	Israel	34	Retrospective	Open	-	-	-	-
Jaen-Torrejimeno (2019)[54]	Spain	105	Retrospective	Open	27	1	3	4
Chopra (2018)[43]	India	40	Prospective	Laparoscopic	40	-	-	40
Acharya (2018)[55]	India	62	Prospective	Open	-	-	-	-
El Gendi (2018)[56]	Egypt	54	RCT	Open	-	-	-	-
Minaev (2017)[57]	Russia	81	Prospective	Open and laparoscopic	39	17	167	-
Duta (2016)[50]	Romania	59	Retrospective	Laparoscopic	-	-	-	59
Abdelraouf (2015)[58]	Egypt	17	Retrospective	Open	-	-	-	3
Georgiou (2015)[59]	Greece	145	Retrospective	Open	18		15	112
Jabbari Nooghabi (2015)[60]	Iran	71	Prospective	Open and laparoscopic	-	-	-	73
Yucel (2015)*[61]	Turkey	438	Retrospective	Open	15	-	-	423
Surmelioglu (2015)[62]	Turkey	186	Prospective	Open	72	-	-	81
Samala (2014)[44]	India	32	Prospective	Laparoscopic	32	-	-	-
Pandey (2014)[63]	India	30	Retrospective	Open and laparoscopic	30	-	-	-
Mehrabi Bahar (2014)[64]	Iran	155	Retrospective	Open	-	-	90	155
Baraket (2014)[65]	Tunisia	120	Retrospective	Open	48	-	-	120
El Malki (2014)[66]	Morocco	407	Retrospective	Open	-	-	-	-
Zeybek (2013)[67]	Turkey	210	Retrospective	Open	-	-	-	-
Maoz (2013)[68]	Israel	29	Retrospective	Open	-	-	-	-
Rooh Ul (2011)[69]	Pakistan	43	Prospective	Laparoscopic	-	-	-	-
Gupta (2011)[34]	India	33	Retrospective	Open	-	-	-	-
Motie (2010)[70]	Iran	71	Retrospective	Open	-	-	-	135
Losada Morales (2010)[71]	Chile	110	Prospective	Open	110	-	-	81
El Malki (2010)[72]	Morocco	47	Retrospective	Open	-	-	-	-
Topcu (2009)[73]	Turkey	30	Prospective	Open	-	-	-	-
Unalp (2009)[74]	Turkey	183	Retrospective	Open	38	-	-	183
Gourgiotis (2007)[75]	Greece	137	Retrospective	Open	72	-	2	43
Fahim (2007)[76]	Saudi Arabia	54	Prospective	Open	-	-	-	-
Manouras (2007)[77]	Greece	5	Prospective	Open	-	-	-	5
Safioleas (2006)[78]	Greece	153	Retrospective	Open	153	-	-	-
Palanivelu (2006)[79]	India	66	Prospective	Laparoscopic	-	55	-	-
Ozmen (2006)[80]	Turkey	20	Prospective	Open	-	-	-	20
Chen (2006)[81]	China	76	Prospective	Laparoscopic	-	-	-	76
Wang (2006)[82]	China	20	Retrospective	Open	-	-	-	-
Elsebaie (2006)[83]	Egypt	32	RCT	Open				
Abbas (2006)[84]	Egypt	18	Prospective	Open	22	-	0	2
Bulbuller (2006)[85]	Turkey	49	Retrospective	Open	15	-	25	41
Mirelis (2006)[86]	Greece	59	Retrospective	Open	37	-	-	18
Kouraklis (2005)[87]	Greece	62	Prospective	Open		-	-	-
Dervisoglu (2005)[88]	Turkey	12	Retrospective	Laparoscopic	33	2	-	12
Dosios (2003)[89]	Greece	8	Prospective	Open	-	-	-	-
Agaoglu (2003)[90]	Turkey	82	Retrospective	Open	16	-	28	54
Mueller (2003)[91]	Germany	13	Retrospective	Open	-	-	-	-
Kurt (2003)[92]	Turkey	7	Retrospective	Open	-	-	-	-
Celebi (2002)[93]	Turkey	49	Retrospective	Open	-	-	-	-
Haddad (2001)[94]	Lebanon	23	Retrospective	Open	-	-	-	-
Cirenei (2001)[95]	Italy	134	Retrospective	Open	-	20	-	-
Prousalidis (1999)[96]	Greece	75	Retrospective	Open	4	-	6	60
Ertem (1998)[97]	Turkey	12	Prospective	Open	3	-	-	-
Vagianos (1995)[98]	Greece	67	Prospective	Open	22	-	-	67
Joshi (1991)[99]	India	9	Retrospective	Open	-	3	3	3

Abbreviations: RCT: Randomized controlled trial.

* Study has reported the number of cysts, instead of number of patients.

### Demographic and baseline characteristics

Most of the 54 included studies reported on patients from the Middle East, North Africa, Greece, and the Indian peninsula. Abdominal pain or right upper quadrant pain were the most common symptoms, followed by nausea, jaundice, and fever. The demographic data of the patients enrolled in included studies, such as age and gender, are provided in S2 Table. The utilization of laboratory tests for diagnosing hepatic CE infection was described in 22 studies (40.7%). However, only 14 studies described the type of serological tests (25.9%); these tests were the Casoni test (four studies, 7.4%), an assay providing an antibody titer (five studies, 9.3%), and an ELISA assay (five studies, 9.3%). Imaging techniques utilized to diagnose the hepatic CE in patients were reported in 48 studies (88.9%), including ultrasonography (43 studies, 79.6%), CT scan (39 studies, 72.2%), X-ray radiography (ten studies, 18.5%), and MRI (five studies, 9.2%). Data of preoperative diagnostic modalities are summarized in S2 Table. Regarding the further preoperative diagnostic and interventions, two studies (3.7%) reported on patients with cholangitis who underwent magnetic resonance cholangiopancreatography and six studies (11.1%) reported on patients who underwent endoscopic retrograde cholangiopancreatography. Although we could not extract the number of patients, cholangitis was considered the main indication for the preoperative interventions.

Cysts were classified according to the Gharbi classification in 15 studies (27.8%) and according to the WHO classification in six studies (11.1%); no classification was reported in 33 studies (61.1%). However, the distribution of the different cysts was only described in 11 studies (eight studies with Gharbi classicifcation and three studies with WHO classification). In addition, three studies reported the staging according to the number of cysts in each category, whereas eight studies reported the number of patients with respective cyst categories. Patients with stage CE2 (41%), CE1 (31.2%), and CE3 (27.8%) cysts according to the WHO classification were the most common candidates for endocystectomy. In studies using Gharbi classification, stage III (32.8%), stage I (25.1%), and stage IV (14.8%) cysts were the most frequent types that were treated by endocystectomy. The detailed staging of the cysts and patients according to the WHO and Gharbi classifications is listed in S3 Table. In one study (1.8%), no medication was prescribed before surgery. The administered drugs included albendazole (31 studies, 100%) and a mixed regimen of albendazole and mebendazole (three studies, 9.7%). Medical treatment was administered preoperatively and was continued postoperatively. In the 21 studies reporting the duration of treatment, albendazole was administered within 1 day to 12 weeks before surgery–two weeks before surgery in most studies (seven studies, 33.3%). Dosages ranged from 10 to 15 mg/kg per day. However, in two studies, patients received 400 mg albendazole twice daily, and in one study, 200 mg albendazole was prescribed twice daily. Of three studies administrating mebendazole, only one reported the dosage, which was 30 mg/kg daily. In 25 studies (46.3%), the method of administration of anthelmintic therapy was reported, which was continuous in 22 (88%) and cyclic in 3 (12%) studies. The detailed description of the preoperative diagnostic approaches, interventions, and medical therapies is summarized in S2 Table.

### Surgical technique

All studies reported using an endocystectomy technique, 11 of which were laparoscopic and 47 of which were open. Modifications to the surgical technique were described in 35 studies (64.8%) as follows: partial cystectomy in 18 studies (33.3%), deroofing in 13 studies (24%), and cystopericystectomy in four studies (7.4%). In all studies, surgeons surrounded the cysts with sterilized saline-soaked gauze to prevent contamination of surrounding tissue with cyst contents. Afterward, they placed a cannula (of varied size) on the most superficial surface of the cyst and aspirated the cyst contents until cysts were evacuated entirely. Subsequently, a scolicidal agent was injected into the cyst cavity to sterilize the cysts and to kill the parasite. Thirteen studies (24.1%) injected a scolicidal agent intraoperatively: 20% NaCl solution in 11 studies (20.3%), chlorhexidine 0.05% in two studies (3.7%), and Savlon 2% (cetrimide and chlorhexidine digluconate) in one study (1.8%). All injections were performed in a single attempt except in the study of Abbas et al., who applied the same scolicidal agent into the hepatic cyst twice. The duration of contact of scolicidal agents with the germinal layer differed between studies. Surgeons waited for 5 minutes in five studies (9.2%), for 10 minutes in five studies (9.2%), for 15 minutes in two studies (3.7%), and for 20 minutes in one study (1.8%). Management of the residual cavity was described in 30 studies (55.5%). These management techniques included drainage in 1,903 patients (49.5%), omentoplasty in 878 patients (22.8%), capittonage in 333 patients (8.6%), marsupialization in 104 patients (2.7%), introflexion in 36 patients (0.9%), and hepatoplasty in five patients (0.1%).

### Surgical outcomes

#### Intraoperative complications

**Fig 2:**
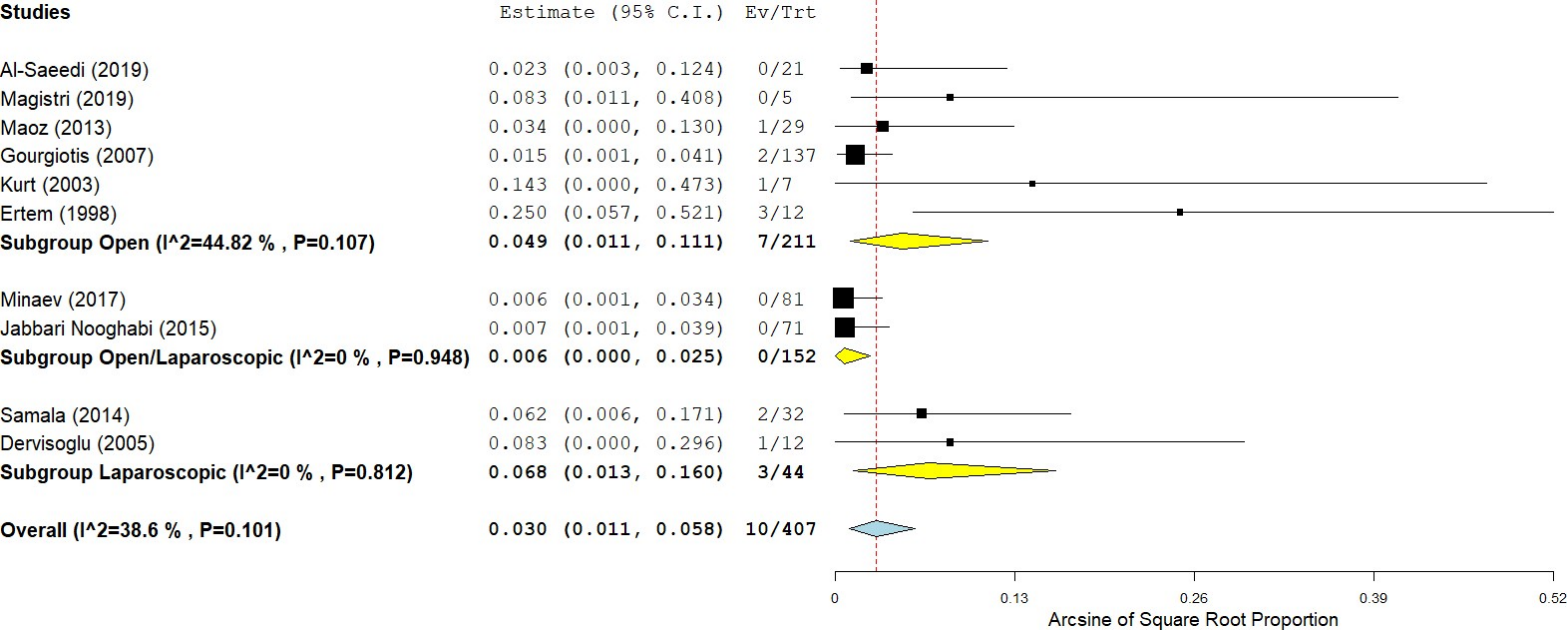
Forest plot of the intraoperative complications rate in endocystectomy for cystic echinococcosis, with subgroup analysis of laparoscopic and open surgeries.

Of 54 studies, intraoperative complications of endocystectomy were reported in eight studies (14.8%) (Fig 2). Intraoperative anaphylactic shock was reported in three out of 641 patients (1.1%; 95% CI: 0.4–2.0; I^2^ = 0%; p-value = 0.96) who underwent endocystectomy with various modifications, including cystostomy, cystopericystectomy, partial cystectomy, and laparoscopic endocystectomy. Other intraoperative complications (such as bleeding, cyst spillage, and bile leakage) were reported in 3% of patients (95% CI: 1.1–5.8; I^2^ = 39%; p-value = 0.10). Intraoperative cyst rupture was reported in two out of 2937 patients (0.06%) included in 38 studies.

#### Postoperative complications and mortality

**Fig 3:**
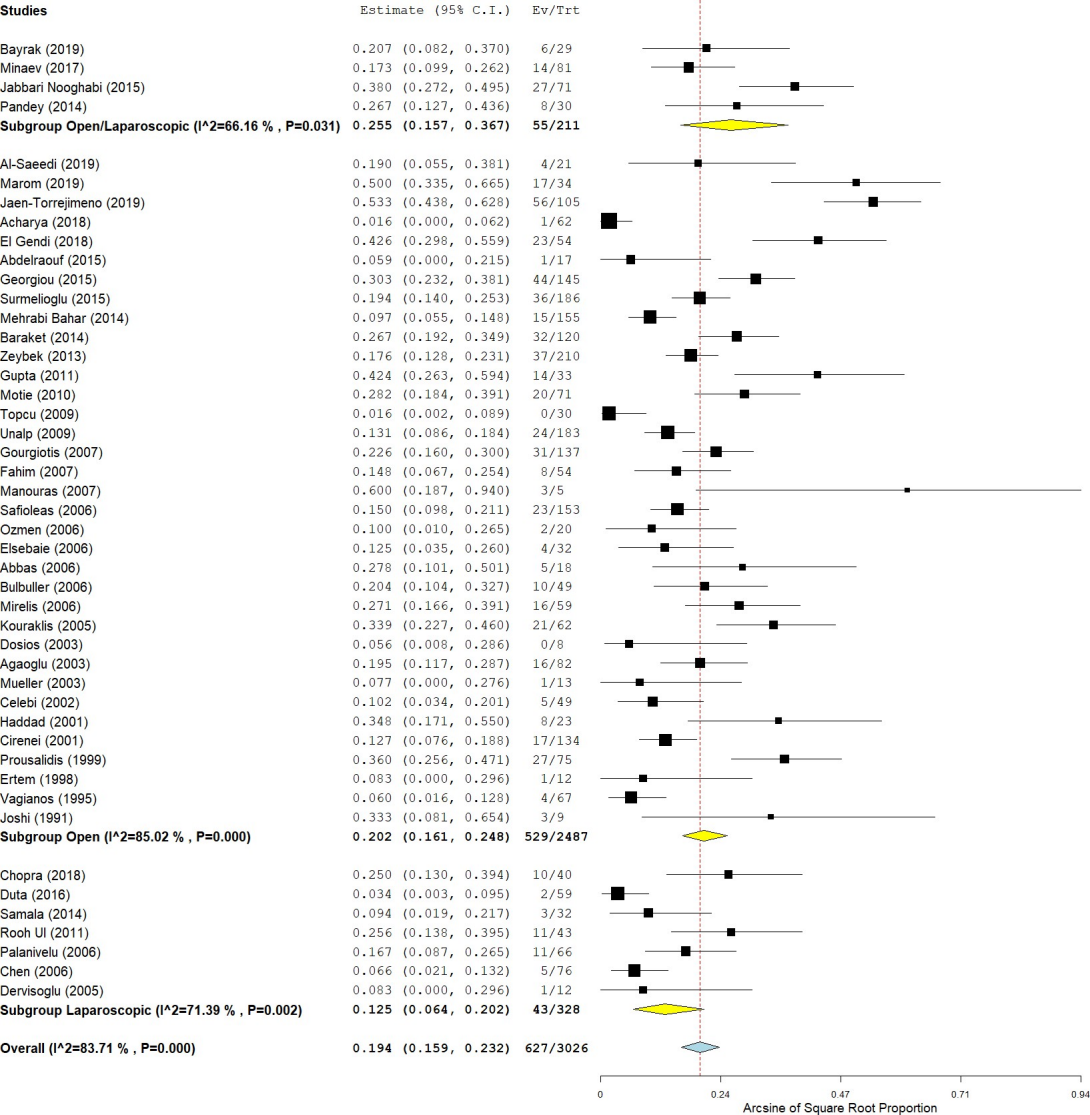
Forest plot of the postoperative complication rate after endocystectomy for cystic echinococcosis, with subgroup analysis of laparoscopic and open surgeries.

As shown in S4 Table, postoperative complications were reported in 47/54 studies (87%). In these studies, postoperative complications occurred in 19.4% of the patients (95% CI: 15.9–23.2; I^2^ = 84%; p-value <0.001) (Fig 3). Rate of postoperative complications in open and laparoscopic approaches was 20.2% (529/2487; 95% CI: 16.1–24.8; I^2^ = 85%; p-value <0.001) and 12.5% (43/328; 95% CI: 6.4–20.2, I^2^ = 71%; p-value = 0.002), respectively. The analysis revealed a significantly lower postoperative complication rate among patients who underwent endocystectomy using a laparoscopic approach compared with an open approach (p-value <0.001).

Postoperative anaphylactic shock was reported in eight out of 641 patients (1.6%; 95% CI: 0.8–2.7, I^2^ = 0%; p-value = 0.71). Biliary leakage was reported in 361 of 2757 patients (10.1%; 95% CI: 7.5–13.1 I^2^ = 81%; p-value <0.001), followed by residual cavity infection in 55 of 1106 patients (4.6%; 95% CI: 3.1–6.4; I^2^ = 35%; p-value = 0.08), and wound infection in 49 of 702 patients (6.6%; 95% CI: 4.6–9; I^2^ = 27%; p-value = 0.17). Only five studies with 169 patients classified postoperative complications based on the Clavien–Dindo classification. In these studies, only 12 patients (7.1%) had major complications (≥ grade IIIa).

Complications were classified according to residual cavity management in 18 studies (33.3%). Complication rates were higher in the marsupialization group (10/23 patients; 43.5%), followed by the capittonage group (18/71 patients; 25.3%), the drainage group (70/317 patients; 22.1%), and the omentoplasty group (69/366; 18.8%). The complication rate was 17.5% in the group with no cystic residual cavity management (41/234 patients). Chi-square analysis revealed a statistically significant difference between different techniques of residual cavity management (p-value = 0.02). Further post-hoc analysis showed no significant correlation between postoperative complications and residual cavity management techniques. Marsupialization showed a non-significant trend for a higher complication rate compared with other techniques (post-hoc p-value = 0.006; significance level after Bonferroni correction <0.005).

**Fig 4:**
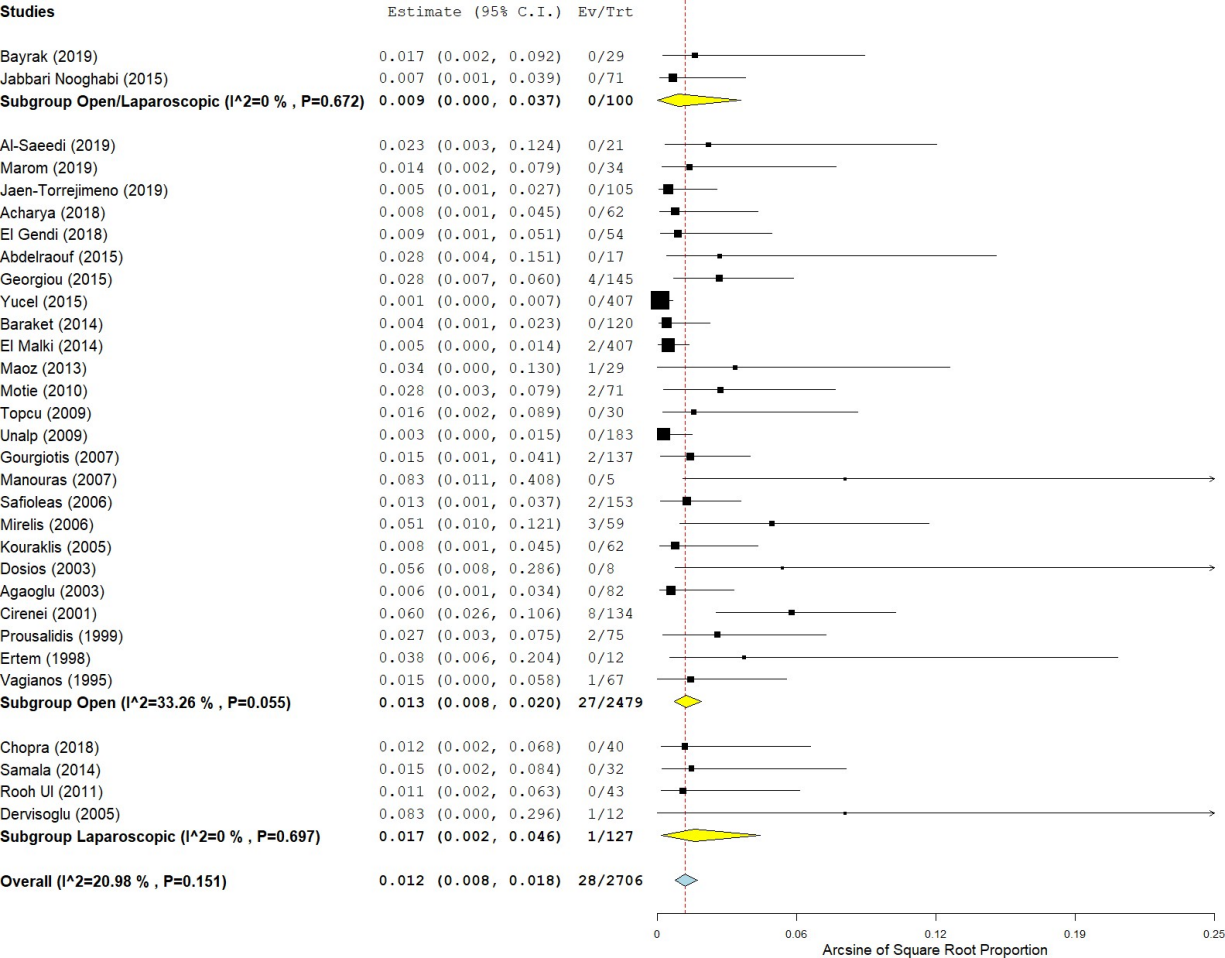
Forest plot of the mortality rate after endocystectomy for cystic echinococcosis, with subgroup analysis of laparoscopic and open surgeries.

The post-endocystectomy mortality rate was reported in 40 studies (74%) including 2706 patients. The all-cause mortality was 1.2% (95% CI: 0.8–1.8 I^2^ = 21%; p-value = 0.15) (Fig 4). Of these, the cause of death was not described in ten patients (35.7%), eight patients died of septic shock (28.5%), three patients died of hemorrhage (10.7%), two patients died of myocardial infarction (7.1%), one patient died of cardiogenic shock (3.6%), one patient died of a cerebrovascular accident (3.6%), one patient died from acute cholangitis (3.6%), one patient died of cerebral hydatiosis and multiorgan failure (3.6%), and one patient died of hepatorenal syndrome (3.6%) (S5 Table). The mortality rates following open and laparoscopic approaches were 1.3% (27/2479; 95% CI: 0.8–2; I^2^ = 33%; p-value = 0.05) and 1.7% (1/127; 95% CI: 0.2–4.6 I^2^ = 0%; p-value = 0.69), respectively. The analysis revealed no significant difference in mortality after open and laparoscopic endocystectomy (p-value = 0.60). The mortality rate was 0.8% in patients who received solitary endocystectomy without any further manipulation. Patients who underwent omentoplasty and capitonnage showed similar mortality rates of 1.7% and 1.8%, respectively. Patients whose residual cavity was managed by drainage implantation had the lowest mortality rate (0.4%). No significant difference was detected between the mortality rates based on the residual cavity management technique (p-value = 0.55).

#### Follow-up and recurrence

**Fig 5:**
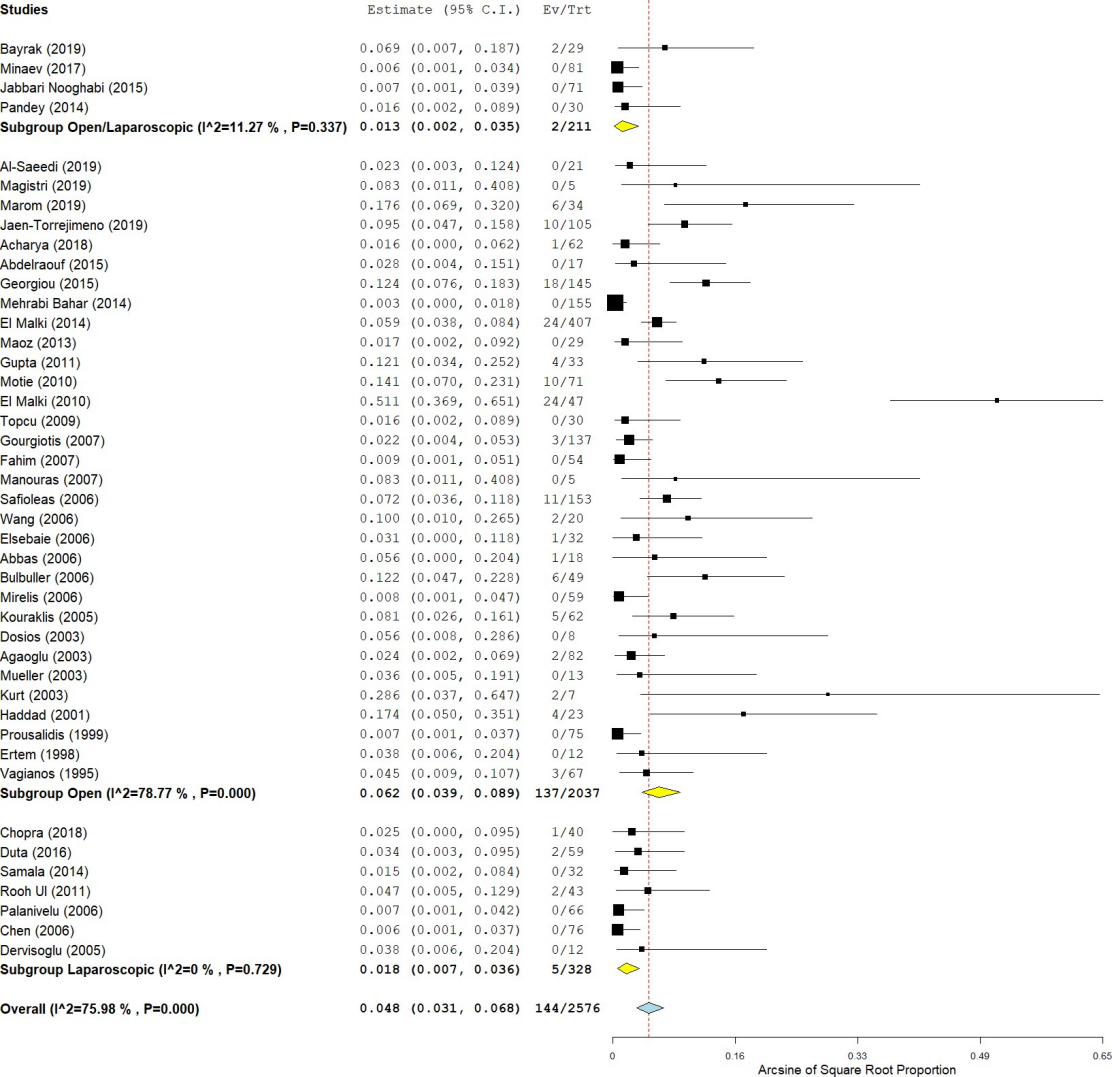
Forest plot of the recurrence rate after endocystectomy for cystic echinococcosis, with subgroup analysis of laparoscopic and open surgeries.

Long-term follow-up after endocystectomy was reported in 44 studies (81.4%). The postoperative follow-up period ranged from six months to ten years, and was between 12 and 60 months in most studies. With due attention to the mean duration of follow-up, 39 studies (88.7%) had a mean follow-up of more than one year after endocystectomy, and only 14 studies (31.8%) had a follow-up of more than five years (S6 Table). Hepatic CE recurrence was reported in 2576 patients from 40 studies (63.4% of total patients). Hepatic CE recurrence occurred in 144/2576 patients (4.8%; 95% CI: 3.1–6.8; I^2^ = 87%; p-value <0.001) (Fig 5). Recurrence rates after open and laparoscopic approaches were 6.2% (137/2037; 95% CI: 3.9–8.9; I^2^ = 79%; p-value < 0.001) and 1.8% (5/328; 95% CI: 0.7–3.6; I^2^ = 0%; p-value = 0.72), respectively. The recurrence was significantly lower in patients who underwent laparoscopic endocystectomy compared with open endocystectomy (p-value <0.001). The recurrence rate was 5.3% (3/56 patients), 4.3% (13/303 patients), and 2.4% (7/286 patients) after endocystectomy with capittonage, omentoplasty, and drainage, respectively. The analysis showed no significant difference between various techniques of residual cavity management (p-value = 0.36).

## Discussion

Despite reports of elimination in remote island countries, CE still represents a healthcare burden [16, 17]. The WHO has listed CE as a neglected disease that should be controlled or eliminated in the next decades [17, 18]. Patients with hepatic CE have a higher morbidity and mortality rate if adequate care is not provided [19]. According to the literature, the current global burden of CE is estimated to be 285,500 disability-adjusted life years (DALYs) (95% CI: 218,515–366,133) or an annual loss of USD 194,000,000 [20, 21]; however, the specific burden of hepatic CE has not been reported. Three main treatment approaches are available for patients with hepatic CE: medical, surgical, and percutaneous [6, 22]. The ultimate goal of all three approaches is to eliminate the germinal layer of the cysts and to prevent recurrence [4]. With due attention to disease burden, choosing the appropriate treatment can cure hepatic CE and decrease the costs associated with morbidity and mortality.

Hepatic CE is primarily diagnosed via imaging techniques; ultrasonography (US) is a relatively inexpensive tool for diagnosis and screening or follow-up [23]. US can identify the specific presentation of hepatic CE and has revolutionized its diagnosis [24]. It provides the opportunity to evaluate all cyst features, including size, presence of daughter cysts and their agglomeration, and calcifications of the cystic wall [25]. US can also be used for cyst staging and treatment planning, which is particularly beneficial in cases of hepatic CE. MRI can also diagnose CE with acceptable accuracy if US examination is not possible because of cyst location or patient-specific reasons, and was found to be superior to CT scans [24, 26, 27]. In our systematic review, 88% of included studies used US to diagnose hepatic CE. Only five of the included studies used MRI to evaluate hepatic CE cysts and 39 used a CT scan. This finding shows that current diagnostic approaches for hepatic CE, particularly the limited use of MRI, need to be reevaluated.

The WHO has classified hepatic CE based on ultrasonographic features of the cyst [6]. This approach is more accurate than previous classification methods since it defines the best treatment strategy and reduces complications. However, this classification system was only used in 12% of the studies included in this systematic review that were published after the guidelines were introduced in 2003. This result is comparable with values reported in another systematic review [28]. According to the WHO, treatment of uncomplicated hepatic CE should be decided based on the cyst stage [6, 29, 30]. On this basis, a watch and wait approach has been suggested for stage CE4 and CE5 cysts [6] while medical treatment is recommended for stage CE1, CE2, CE3a, and CE3b cysts [6]. Surgical treatment is limited to stage CE2 and CE3b cysts while percutaneous treatments can be performed for stage CE1, CE2, CE3a, and CE3b cysts [6].

Before anthelmintic agents were introduced, surgery was the treatment of choice in patients with hepatic CE. Despite the low morbidity and mortality, radical surgeries might not be applicable in all cases [31–33]. These drawbacks have forced physicians to introduce less harmful and more accurate treatment options. However, invasive surgery is still sometimes needed to eradicate parasitic infection in patients with complicated hepatic cysts or who do not respond to anthelmintic therapy. In recent years, conservative treatment has become more acceptable among surgeons [34, 35]. In contrast to radical techniques, which can include cystectomy and removal of the germinative layer by non-anatomical liver resection, conservative interventional and surgical procedures aim to eliminate cyst materials while leaving the germinative layer intact. Our current comprehensive literature search revealed that endocystectomy has been used to address a conservative surgical treatment for hepatic CE. Conservative techniques have included different surgical approaches, such as endocystectomy, external drainage, the Mabit procedure (deroofing the cyst and extracting the parasite), partial pericystectomy (leaving a large piece of cyst wall deep within the liver), and subtotal pericystectomy [36]. In an international consensus on echinococcosis terminology Vuitton et al., defined partial cystectomy as an endocystectomy [37]. In our review, we considered "endocystectomy" to be evacuation of the cyst content and partial removal of the cyst wall, which reflects the advantages of endocystectomy over other conservative approaches.

The WHO has recommended the puncture, aspiration, injection, and respiration (PAIR) technique for treating patients with stage CE1 and CE3a hepatic CE (cysts < 10 cm in size) [3, 38–40]. Although PAIR has diagnostic and treatment benefits, it can only be used to treat stage CE1 or CEa hepatic CE [34]. In contrast, endocystectomy can be used to treat cysts of any stage, although the criteria for endocystectomy remain controversial among experts [35]. Our review revealed that endocystectomy has been used to treat hepatic cysts of all stages, although mainly stages CE2, CE1, and CE3 were treated by endocystectomy. Although we could not compare cyst stages operated by open and laparoscopic endocystectomy, the analysis showed a lower postoperative complication rate and recurrence following laparoscopic endocystectomy. In addition, the laparoscopic approach can directly visualize the cyst cavity to observe the cystobiliary communication and remnant germinal layer [41]. These advantages together with better cosmetic results, decreased hospital stay, and reduced analgesic requirement make laparoscopic an encouraging option for hepatic CE treatment [42–44].

According to our findings, some modifications have been recommended to manage the residual cavity after hepatic CE treatment via endocystectomy. Our analysis showed no significant differences in complications and CE recurrence between the different approaches, suggesting the management procedure should be chosen at the surgeon’s discretion. Akhan et al. have also reported a modified catheterization technique for removing cyst contents in patients with CE2 and CE3b cysts, with encouraging outcomes and a low recurrence rate [45]. The procedure is suggested to be safe and effective, but the risk of repetitive intervention if the cyst is not completely removed can be considered a weakness. Their primary study illustrated promising advantages of the technique, which can outscore the disadvantages; nonetheless, further studies are needed to confirm its efficacy.

Although some studies have claimed that postoperative complications such as biliary fistula and abscess formation are less prevalent following radical surgical treatments, comparable complication rates have been reported following conservative methods such as endocystectomy [5, 46]. In this study, we aimed to assess the overall complication, mortality, and recurrence rate in patients following endocystectomy to treat hepatic CE. We found a morbidity rate of 19.4% in patients receiving endocystectomy, which was similar to the morbidity rate following PAIR in a meta-analysis by Sokouti et al. (18.5%) [47]. The frequency of postoperative complications following radical surgery for hepatic CE was higher than the frequency of complications following conservative surgeries for hepatic CE in a meta-analysis [46]. In another study, the morbidity rate was 25.1% following surgical intervention and 7.9% following PAIR [48]. In the present study, we calculated a postoperative mortality rate of 1.2% among 2706 patients; this was comparable to the 1.1% mortality rate following PAIR that was reported in another meta-analysis [47]. Another study reported a mortality rate of 0.1% following conservative surgeries for hepatic CE, including PAIR and endocystectomy [48]. In this study analysis, authors reported a hepatic CE recurrence rate of 1.5% following conservative surgery and 6.3% following radical surgery. However, the recurrence rate after the PAIR procedure was between 5% and 6.6% [36, 47, 48]. Our results revealed a recurrence rate of 4.8% in 2576 patients treated with endocystectomy. Although recurrence rates varied widely between 0% and 51% (with most between 0% and 15%) in the included studies, 19/54 studies reported 0% recurrence during follow-up. These findings suggest that endocystectomy may be safe and effective; however, the reported outcomes probably depended on the duration of follow-up and perioperative treatment with albendazole. The data we extracted from the literature did not show the correlation of pre- and postoperative anthelmintic treatment with CE recurrence. In addition, our results do not support a correlation between intraoperative cyst rupture and CE recurrence. Of four studies reporting patients with cyst rupture, only in one study recurrence was reported after cyst rupture.

Our results suggest that, although endocystectomy has a slightly higher morbidity rate, it has similar mortality and recurrence rates to PAIR and radical surgeries. Although the WHO recommend PAIR to have beneficiary outcomes in treatment of hepatic CE patients, it can only be used in patients with stages CE1 and CE3a of hepatic CE [3, 38–40]. Radical surgeries such as hepatectomies have a high risk of postoperative complications and prolonged hospital stay [34, 49] so endocystectomy is more agreeable treatment option with acceptable outcomes for hepatic CE patients who do not meet the criteria for PAIR.

To our knowledge, this is the first study to systematically review the literature to evaluate the advantages and disadvantages of the endocystectomy technique. However, there are limitations to our study. Since we aimed to comprehensively summarize the current knowledge based on the literature, we did not wish to restrict our search and exclude old studies. The main weaknesses are the low quality of the included studies and heterogeneity between studies. Furthermore, because well-designed studies are lacking, we decided to include all eligible articles. However, there was undeniable controversy between the results of the included studies, probably because of heterogeneity in study quality and design. This emphasizes the need for well-structured research in this area. In some studies, data were not unified or well-described and could not be extracted for analysis. It was not possible to analyze quantitative variables, such as intraoperative blood loss and hospital stay. In addition, the recurrence rate was not reported or the follow-up was not long enough to calculate the recurrence rate over time in some studies. In their study, Duta et al., reported the outcomes of endocystectomy in 59 patients, including 17 patients with CL stage CE cysts [50]. Since the outcomes were not reported separately for each cyst stage, we could not exclude patients with CL-stage cysts from the analysis, which could also lead to bias in our analysis of CE recurrence.

In conclusion, our results suggest that endocystectomy has promising outcomes in the treatment of hepatic CE. It can be considered an effective and safe alternative in patients with cysts that cannot be cured with the PAIR procedure. Surgeons should consider endocystectomy as a parenchyma-sparing technique that avoids radical resections. We believe randomized trials are needed not only to compare endocystectomy with other conservative techniques or radical surgical approaches but also to compare different modifications of endocystectomy.

## Supporting information

S1 Table **(a)** Assessment of the quality of included studies according to MINORS. **(b)** Assessment of the quality of randomized controlled trials according to Cochrane Risk of Bias Tool for Randomized Controlled Trials.
(XLSX)

S2 Table Demographic data of the included patients, preoperative diagnostic workups, and anthelmintic treatments reported in the studies. (XLSX)

S3 Table Stage of the operated cysts by endocystectomy according to WHO and Gharbi classification, as well as the intraoperative rupture of the cysts. (XLSX)

S4 Table Overall postoperative complication-, mortality-, and recurrence rates. (XLSX)

S5 Table Reported causes of mortality in patients undergoing endocystectomy. (XLSX)

S6 Table Postoperative prophylactic anthelmintic treatment, follow-up details, and recurrence event after endocystectomy. (XLSX)
